# Circulating microparticles are increased amongst people presenting with HIV and advanced immune suppression in Malawi and correlate closely with arterial stiffness: a nested case control study

**DOI:** 10.12688/wellcomeopenres.17044.2

**Published:** 2022-10-17

**Authors:** Christine Kelly, Rijan Gurung, Raphael Kamng'ona, Irene Sheha, Mishek Chammudzi, Kondwani Jambo, Jane Mallewa, Alicja Rapala, Rob Heyderman, Patrick Mallon, Henry Mwandumba, Saye Khoo, Nigel Klein

**Affiliations:** 1 Centre for Experimental Pathogen Host Research, UCD, Dublin, Ireland; 2 Malawi-Liverpool-Wellcome Clinical Research Programme, Blantyre, Malawi, Malawi; 3 Institute of Translational Medicine, University of Liverpool, Liverpool, UK; 4 Institute of Infection, immunity and Inflammation, UCL, London, UK; 5 Liverpool School of Tropical Medicine, LSTM, Liverpool, UK; 6 College of Medicine, University of Malawi, Blantyre, Malawi; 7 Institute of Cardiovascular Science, UCL, London, UK

**Keywords:** HIV, microparticles, cardiovascular

## Abstract

**Background:** We aim to investigate whether circulating microparticle (CMPs) subsets were raised amongst people presenting with a new diagnosis of HIV and advanced immune suppression in Malawi, and whether they associated with arterial stiffness.

**Methods**: Microparticle characterisation and carotid femoral Pulse Wave Velocity (cfPWV) were carried out in a cohort of adults with a new HIV diagnosis and CD4 <100 cells/µL at 2 weeks post ART initiation. HIV uninfected controls were matched on age, systolic BP and diastolic BP in a 1:1 ratio.  Circulating microparticles were identified from platelet poor plasma and stained for endothelial, leucocyte, monocyte and platelet markers.

**Results:** The median (IQ) total CMP count for 71 participants was 1 log higher in HIV compared to those without (p<0.0001) and was associated with arterial stiffness (spearman rho 0.47, p<0.001). In adjusted analysis, every log increase in circulating particles showed a 20% increase in cfPWV (95% CI 4 – 40%, p=0.02).

In terms of subsets, endothelial and platelet derived microparticles were most strongly associated with HIV. Endothelial derived E-selectin+ CMPs were 1.3log-fold higher and platelet derived CD42a+ CMPs were 1.4log-fold higher (both p<0.0001). Endothelial and platelet derived CMPs also correlated most closely with arterial stiffness [spearman rho: E-selectin+ 0.57 and CD42a 0.56, both p<0.0001).

**Conclusions:** Circulating microparticles associate strongly with arterial stiffness among PLWH in Malawi. Endothelial and platelet microparticles are the predominant cell origin types, indicating that platelet driven endothelial dysfunction pathways warrant further investigation in HIV associated arterial stiffness.

## Introduction

People living with HIV (PLWH) are at increased risk of cardiovascular diseases such as myocardial infarction and stroke ^1– 4^. Heightened inflammation has been implicated in vascular dysfunction but the underlying pathogenetic mechanisms have not been fully elucidated ^2^. These mechanisms are even more complex in the low-income sub-Saharan Africa setting (SSA), where additional factors including latent or recurrent infections, late presentation and ART failure are prevalent ^5– 7^. In Malawi, several studies have shown an increased risk of stroke associated with HIV ^8, 9^.

The first few months of antiretroviral treatment (ART) carry a particularly high cardiovascular risk. In a case control study of 705 patients, the odds of stroke were much higher within the first 6 months of ART compared to those who were treatment naïve (adjusted odds ratio 15.6), as well as in those with CD4 less than 200 cells/mm ^3^ compared to those with CD4 greater than 500 cells/mm ^3^ (adjusted odds ratio 12.9) ^9^. 

Arterial stiffness has been used as a measure of vascular dysfunction and cardiovascular risk in HIV studies as well as other chronic inflammatory conditions ^10, 11^. cfPWV is a gold standard measurement of arterial stiffness and has been validated as a physiological biomarker of cardiovascular events and mortality ^12, 13^. Arterial stiffness is therefore used as a physiological marker of the risk of future cardiovascular events. 

We have previously shown that amongst people presenting with HIV and a CD4 count less than 100 cells/mm ^3^, arterial stiffness is increased by 12% (adjusted fold change) compared to healthy volunteers living without HIV ^5^. However, this effect only persists during the first 3 months of ART and is associated with markers of inflammation, including T cell activation ^5^. Given the timing of this inflammatory process, it is tempting to hypothesise that increased cardiovascular risk during this time may follow a similar process to Immune Reconstitution Inflammatory Syndrome (IRIS). During the normal inflammatory response leucocytes adhere to the endothelial cells via VCAM, ICAM , and undergo transcytosis leading to the development of foam cells ^14^. Pro-thrombotic pathways attract platelets to the resulting plaque and subsequent migration of smooth muscle cells into the intima leads to the formation of a fibrous cap ^15^. During this process, elastin is degraded by enzymes such as MMPs and is replaced by collagen, increasing arterial stiffness ^15^.

Circulating Microparticles (CMPs) are released into the circulation following inflammation driven activation or apoptosis of the affected cells ^16^. Through a process of blebbing, microparticles are formed from the originating host cell’s outer membrane (e.g. leucocytes, platelets and endothelial cells). During this process annexin V molecules, which are normally located on the inner membrane of a cell, are flipped round to become expressed on the microparticle surface ^17^. These microparticles also express the markers expressed on the surface of the cell of origin and so microparticle subsets are an indication of which cells are undergoing stress.

Previous studies in the field of HIV have assessed the presence and function of CMPs amongst PLWH in high resource settings and have separately highlighted tissue factor expression, imbalanced endothelial progenitor cell proportions and platelet activation ^18– 21^. Hijmans *et al.* recently demonstrated evidence of *in vitro* endothelial cell stress, apoptosis and senescence induced by leucocyte, platelet and endothelial microparticle subsets from patients with HIV ^22^. Here, we aim to take the characterisation of circulating microparticles in HIV a step further by assessing their relationship with cardiovascular risk, as measured by arterial stiffness, and by seeking to elucidate pathways that might be involved in heightened inflammation during early ART.

## Methods

### Study cohort

The Study into HIV, Immune activation and EndotheLial Dysfunction (SHIELD) cohort recruited 279 ART-naïve adults with a new HIV diagnosis and CD4 <100 cells/µL from the ART clinic and voluntary HIV testing clinic at Queen Elizabeth Central Hospital, Blantyre, Malawi, along with 110 adults without HIV infection and without evidence of infection within the previous 3 months as determined by the study clinician. This cohort (in which some SHIELD patients were also co-recruited into the Reduction of EArly mortaLITY in HIV-infected adults and children starting antiretroviral therapy (REALITY) clinical trial) has been reported on previously, and full details of recruitment are available in 5. In brief, ART-naïve adults with a new HIV diagnosis and CD4 <100 cells/µL underwent a detailed clinical assessment, blood draw for plasma storage and cfPWV at 2 weeks post ART initiation. Study procedures were carried out a two weeks so as to avoid burdening the clinically vulnerable population at the initial ART visit. cfPWV measured arterial stiffness using a Vicorder device (Skidmore Medical, London, UK) according to standardised guidelines ^23^. A random sample of wave forms was reviewed by an experienced independent assessor at three timepoints during the study to ensure consistent quality.

A convenience sample of 36 participants from the PLWH cohort was chosen in two groups. The first group were those with a cfPWV in the highest quartile (>8.2 m/s), from which 24 participants were randomly chosen. The second were those with a cfPWV in the bottom three quartiles (<8.2m/s), from which 12 participants were randomly chosen. The aim of having a 2:1 ratio between highest cfPWV and lower cfPWV values was to enrich the number of potential CMPs for subset analysis (we hypothesised they would be higher at higher ranges of cfPWV) whilst also capturing a range of values to analyse associations between total CMP count and cfPWV. Participants without HIV were then matched to the selected PLWH participants on age, systolic BP and diastolic BP in a 1:1 ratio.

### Statistical analysis

Wilcoxon Ranksum and spearman rho were used to test association between CMPs and categorical or continuous variables respectively. To allow for multiple comparisons characterising 18 types of CMPs, the Bonferroni correction was applied and a p value of less than 0.003 was used as the level for statistical significance. Linear regression was used to examine the association between total CMPs on cfPWV which was log transformed for normality. The model was adjusted for confounders (age and sex) and mediators (blood pressure, haemoglobin and HIV) as identified previously ^5^.

### Laboratory procedures

***Microparticle isolation.*** Plasma samples collected from 10ml sodium citrate tubes and frozen at -80°C were thawed in a 37°C water bath for 1 minute. 250µL was centrifuged at 5000g for 5 minutes in order to isolate platelet poor plasma (PPP). PPP was centrifuged at 16000g for 60 minutes and the PPP was decanted to leave 20µL of microparticle pellet. Distilled water was filtered through 0.22 um syringe filter under a flow hood was added to Annexin V 10x buffer at a 1:10 dilution. Annexin V 1x buffer was added to the microparticle pellet to a volume sufficient to allow 35µL of microparticle/Annexin V buffer solution for each antibody combination being tested as well as controls.

***Microparticle staining.*** Each antibody was diluted to a 1:100 concentration in either AnV buffer for AnV antibodies or in PBS for all remaining antibodies. 5µL of AnV antibody was added to each well containing 35µL of microparticle AnV buffer solution. The remaining origin stains were then added at a volume of 10µL for those tubes that only had one origin stain and 5µL for those tubes that had two origin stains. This was to ensure a total staining volume of 50µL for all samples. Single stain samples were also acquired for the purposes of compensation and isotype controls were analysed for the purposes of gating. For the isotype controls, 10µL of 1:40 isotype control antibody was added to the 35µL microparticle AnV buffer solution along with 5uL of AnV antibody (IgG1 PE, R&D Systems; IgG1k PE, IgG1 FITC, R&D Systems; IgG1k APC Cy7, BD Pharmingen; IgGMk PE, BD Pharmingen).

Following staining, plates were covered with foil and agitated at room temperature for 20 minutes on a plate shaker. 200µL AnV buffer was added to every well and then transferred to FACS tubes. A further 400µL AnV buffer was then added to every tube. Finally, 6µL of 3µm latex beads (SIGMA) were added to 2ml of distilled filtered water and 10µL of that was added to 650µL distilled filtered water to allow microparticle enumeration. Beads size 1.1µm were used for microparticle gating, with microparticles 1µm or smaller and expressing AnnexinV being categorised as microparticles.

Endothelial, leucocyte, monocyte and platelet microparticles were chosen as common circulating microparticles involved in inflammation and were identified by flow cytometry on a CyAn ADP 9 colour flow cytometer (Beckman Coulter). FITC stained for Annexin V (BD Pharmingen), PE stained for VCAM, ICAM, E-selectin, 66b or CD16 (BD Pharmingen) and APC-Cy7 stained for PECAM or CD14 (BD Pharmingen). To identify tissue factor expression on monocytes, PE stained Annexin V, APC-Cy7 stained CD14 (BD Pharmingen) and FITC stained tissue factor (Sekisui Diagnostics). Single stain samples were also acquired for the purposes of compensation and isotype controls were analysed for gating.

**Figure 1.**
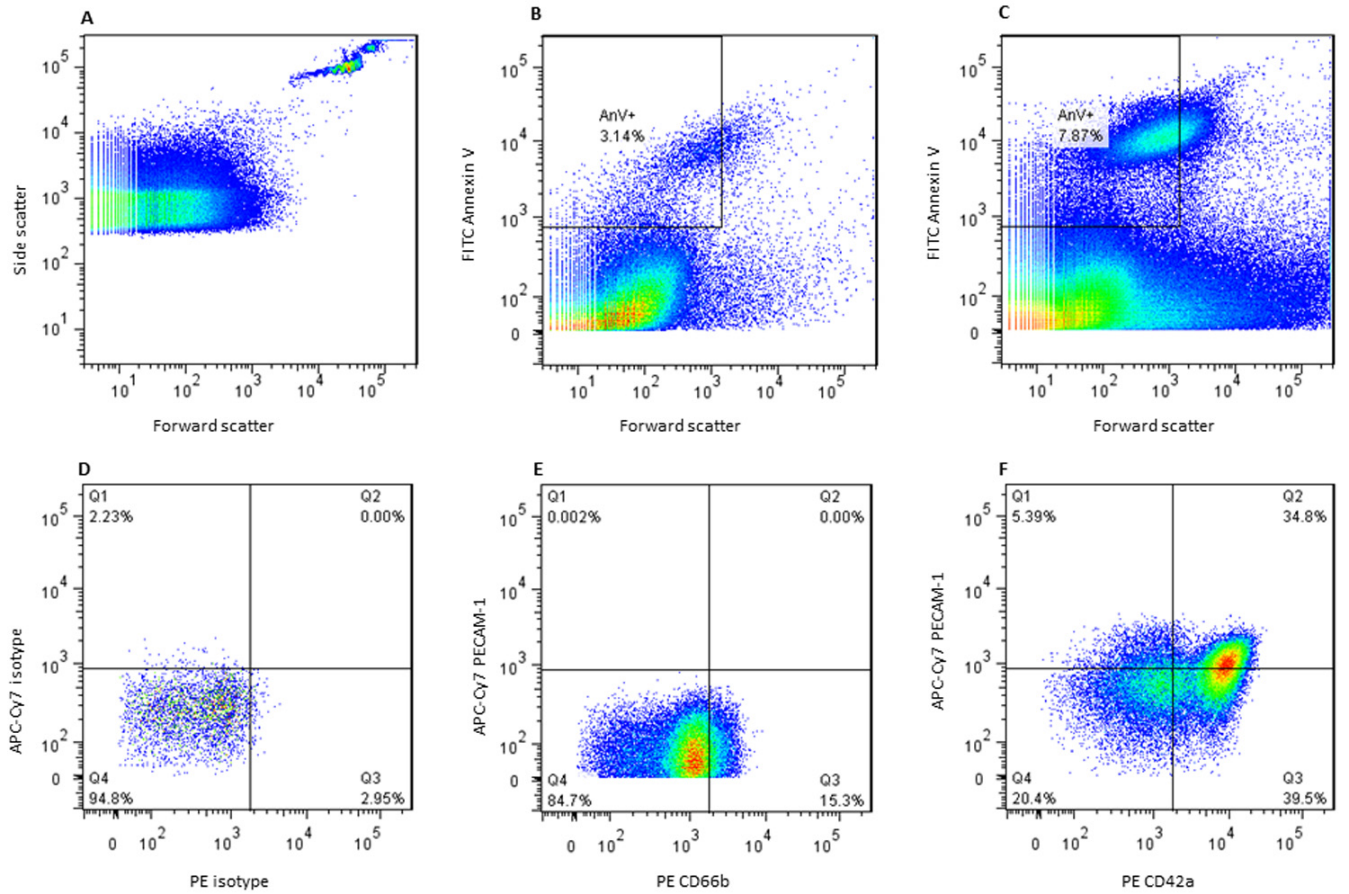
Identification of the microparticle population and subset gating strategy. **A**. 1.1µm beads were used to find forward scatter gate for microparticle identification **B**. Microparticle pellet was obtained from platelet poor plasma and stained for Annexin V. Total circulating microparticle count was measured by identifying microparticles Annexin V positive and less than 1.1µm forward scatter as shown. **C**. Total circulating microparticles in a patient with a high count compared to B. **D**. Isotope controls identified gates for PE and APC-Cy7 stains for each subset panel. **E**. Leucocyte microparticles **F**. Endothelial or platelet microparticles.

***Microparticle gating.*** A microparticle protocol was created on the CyAn flow cytometer with the same voltage settings as the T cell and monocyte panels but with a lower capture threshold of 0.01% instead of 2%. This was to ensure that microparticles were not excluded as debris. 350µL of the contents of each FACS tube was acquired and the plots were then transferred to Flow Jo (Tree star Inc.) for analysis. After identification of singlets, the microparticle pellet was gated on forward scatter and AnV (FITC) to identify the microparticle population which was less than 1µm in size and expressing AnV. Gates were applied using thresholds provided by isotype controls (see Figure 1).

***Cell surface immunophenotyping.*** Cell surface immune phenotyping was carried out for CD4 and CD8 T cell activation (HLA-DR+/CD38), exhaustion (PD1+) and senescence (CD57+), as well as monocyte subsets (classical CD14 ^++^CD16 ^-^, intermediate CD14 ^++^CD16 ^+^ and nonclassical CD14 ^+^CD16 ^+^) as described previously ^5^. The T-cell panel consisted of CD3 BV510, CD4 V450, CD38 PE Cy7, HLA-DR AF700, PD1 APC, and CD57 FITC (all from BD Biosciences) and CD8 PE (Biolegend). The monocyte panel consisted of HLA-DR AF700, CD14 PE Cy7 and CD16 PE (all from BD Biosciences).

### Ethical considerations

Ethical approval for the study was obtained from College of Medicine Research and Ethics Committee (COMREC) of the University of Malawi (P.09/13/1464) and from the University of Liverpool Research and Ethics Committee (UoL000996). Written informed consent was obtained from each participant before taking part in the study.

## Results

CMP data were available for 33 PLWH and 36 people without HIV. Sufficient plasma samples were not available for three of the selected participants with HIV. For the 69 participants with available CMP data, median (interquartile range [IQR]) age was 41 years (35 – 49) and 28 (41%) were female. 31 (45%) had an education level of at least primary school completion. Median (IQR) blood pressure was 130/78 (118/70 – 134/80) and 11 (16%) had a history of smoking. Characteristics according to HIV status are shown in Table 1 (for in depth comparison according to HIV status see 5). For the 33 PLWH, median (IQR) CD4 and HIV viral load were 42 cells/µL (31 – 71) and 1.1×10 ^5^ copies/mL (0.4 – 2.6) respectively.

**Table 1.** Clinical characteristics of 67 SHIELD participants with microparticle data according to HIV status.

	People living with HIV n=33		People living without HIV n=36	
	Median or Frequency	IQR or %	Median or Frequency	IQR or %
**Age (years)**	41	38 – 50	40	34 – 50
**Female**	13	39%	15	42%
**Primary school education or less**	16	48%	15	42%
**Body Mass Index (kg/m ^2^)**	21.7	19.4 – 24.7	22.1	19.4 – 23.8
**Systolic BP (mmHg)**	130	122 – 135	128	118 – 134
**Diastolic BP (mmHg)**	80	74 – 88	76	69 – 79
**Haemoglobin (g/dL)**	12	11 – 13	14	13 – 15
**Cholesterol (mmol/L)**	4.3	3.7 – 4.7	4.2	3.7 – 4.9
**Glucose (mmol/L)**	4.6	4.3 – 5.0	4.6	4.2 – 5.4
**Creatinine (mmol/L)**	68	58 – 83	66	57 – 80
**History of ever smoking**	6	18%	5	14%
**History of a diagnosed cardiovascular disease**	3	9%	4	11%
Co-infection at time of recruitment *	3	9%	1	3%
**CD4 count (cells/µL)**	42	31 - 71	-	-
**HIV Viral Load** **(x10 ^5^ copies/mL)**	1.1	0.4 – 2.6	-	-
**cfPWV (m/s)**	9.1	8.2 – 10.3	8.0	6.7 – 8.7
**Total Circulating Microparticle Count (log particle/mL)**	6.7	6.3 – 7.3	5.6	5.3 – 5.8

*Acute co-infection, refers to diagnosis of an infection other than HIV

### Relationship between absolute CMP counts, HIV and arterial stiffness

**Figure 2.**
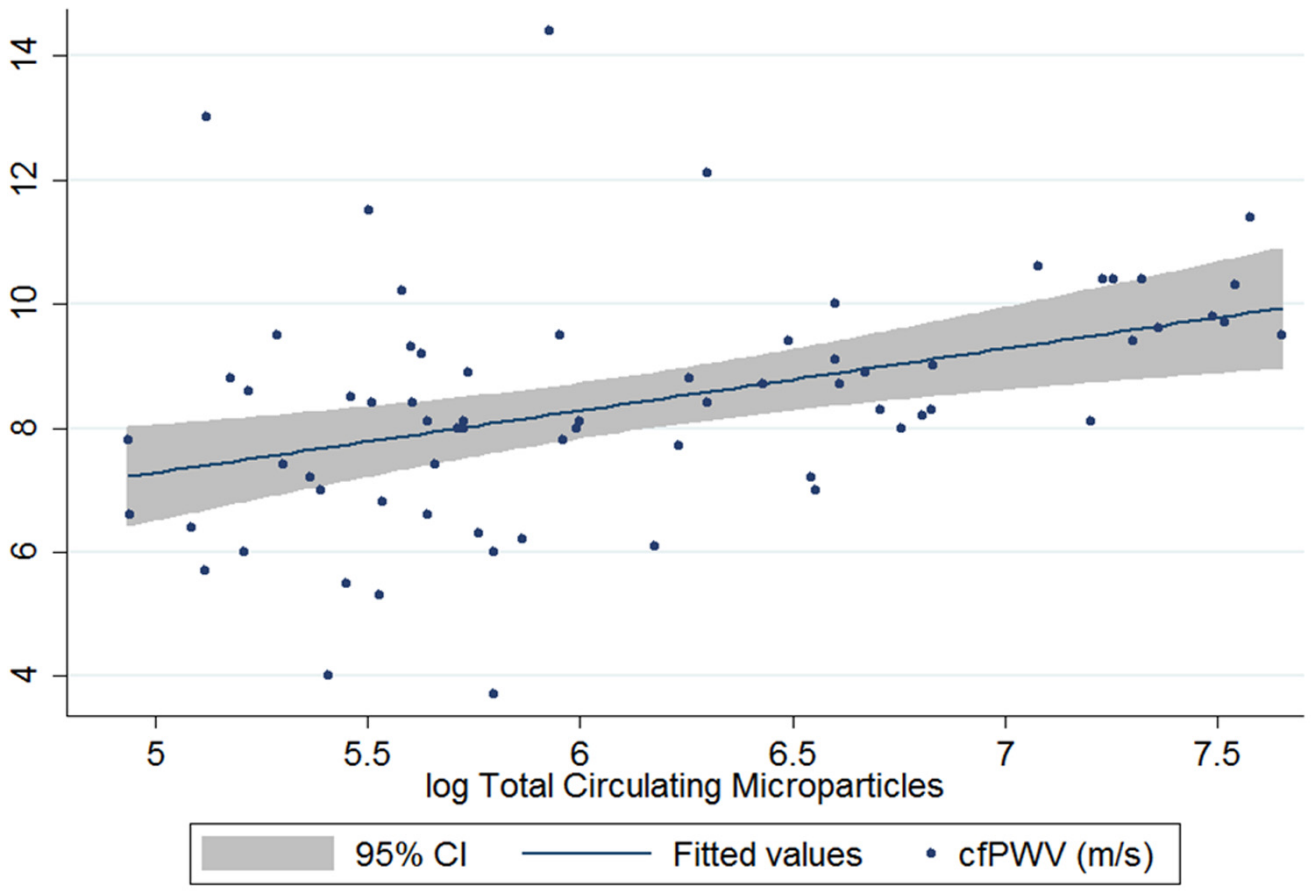
Association between total circulating microparticles and carotid femoral pulse wave velocity (m/s). Correlation analysis demonstrated spearman rho 0.47, p<0.001.

For the overall cohort, the median (IQ) total CMP count was approximately 1 log higher in PLWH compared to those without HIV ( Table 1). Total CMP counts were also significantly associated with arterial stiffness (spearman rho 0.47, p<0.001; Figure 2), as well as faster heart rate and higher creatinine (spearman rho: 0.31; p=0.01 and 0.28; p=0.02). When adjusted for *a priori* identified mediators and confounders (age, sex, haemoglobin and blood pressure), higher log total CMPs were associated with an increased cfPWV [fold change 1.20m/s, 95% CI 1.04 – 1.40; p=0.02]. When additionally adjusted for HIV status, the association between log total circulating microparticle count and cfPWV remained similar [fold change 1.07m/s (95% CI 1.23 – 1.01), p=0.046. Table 2].

**Table 2.** Linear regression analysis showing the association between total circulating microparticles and carotid femoral Pulse Wave Velocity. A multivariate model was constructed to assess the association between circulating microparticles (CMPs) and carotid femoral Pulse Wave Velocity (cfPWV) using data from patients with and without HIV combined. The models are adjusted for mediators and confounders as identified from previous work in this cohort ^5^. The first model provides the association between CMPs and arterial stiffness adjusting for mediators and confounders but excluding HIV. The second model adds HIV to assess for any role of HIV in mediating the association between CMPs and arterial stiffness.

	Association between total circulating microparticle count and cfPWV			Association between total circulating microparticle count and cfPWV adjusting for HIV		
	Fold change	95% CI	P value	Fold change	95% CI	P value
**Total Microparticle Count** **(per log particle/mL increase)**	1.20	1.04 – 1.40	0.02	1.23	1.01 – 1.50	0.046
**Age** **(per 10 year increase)**	1.14	1.03 – 1.26	0.02	1.12	1.02 - 1.24	0.03
**Female sex**	0.85	0.67 – 1.07	0.18	0.84	0.66 – 1.07	0.17
**Diastolic Blood pressure (per ** **10mmHg increase)**	1.20	1.06 – 1.38	0.004	1.21	1.07 – 1.38	0.004
**Haemoglobin** **(per g/dL increase)**	1.04	0.98 – 1.10	0.19	1.03	0.96 – 1.10	0.41
**HIV infection**	-	-	-	0.95	0.66 – 1.35	0.76

Total CMP counts also correlated directly with proportions of CD4 and CD8 T cells expressing markers of activation, exhaustion and senescence [spearman rho (p value): CD8 activation 0.49 (0.0004), exhaustion 0.39 (0.041), senescence 0.44 (0.0006); CD4 activation 0.44 (0.071), exhaustion 0.27 (0.0007), senescence 0.44 (0.01)]. However, there was no association between total CMPs and monocyte subsets [spearman rho (p value): classical 0.08 (0.57), intermediate -0.018 (0.90), nonclassical -0.07 (0.63)].

### Relationship between CMP subsets, HIV and arterial stiffness

**Figure 3.**
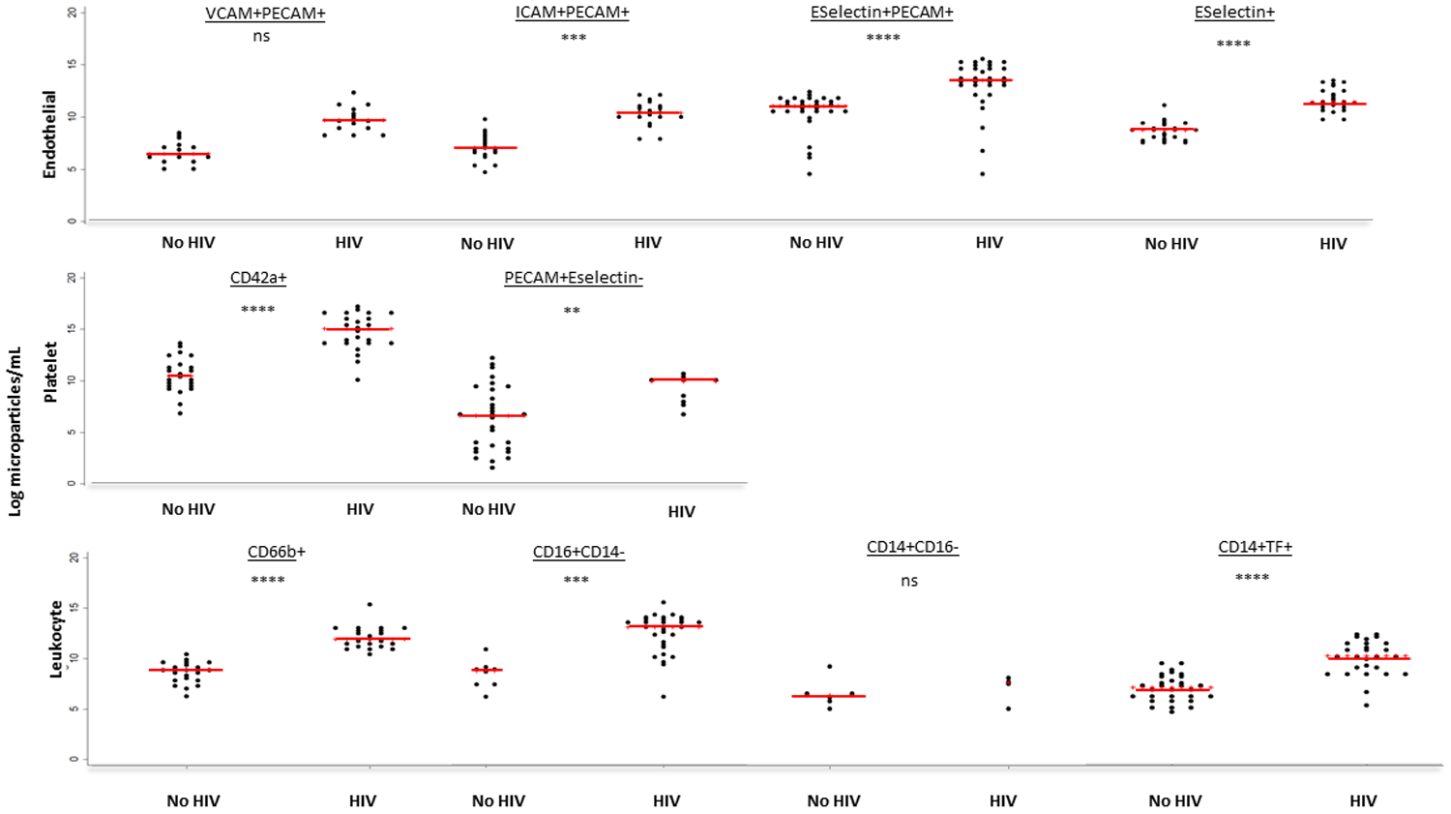
Microparticle subsets according to HIV Status for 69 Malawian Adults. PLWH=People living with HIV. No HIV= People living without HIV. Red lines represent median microparticles count. Grey Asterix represent p value: ns denotes p>0.003, * denotes p<0.003 but >0.0001, **denotes p<0.0001, ***denotes p<0.0001. TF=tissue factor. CD=cluster of differentiation. VCAM=vascular cell adhesion molecule 1. ICAM=intracellular cell adhesion molecule 1. PECAM=platelet endothelial cell adhesion molecule.

The largest elevations in CMP subsets amongst PLWH were seen with endothelial and platelet derived microparticles. Endothelial derived E-selectin+ CMPs were 1.3-fold higher [median (IQR) 13.0 log particles/mL (12.5 – 14.3) vs 9.9 (8.9 – 10.5); p<0.0001]. The increase in CMPs expressing PECAM but not E-selectin was less significant ( Figure 3). Platelet derived CD42a+ CMPs were 1.4-fold higher amongst PLWH [median (IQR) CD42a 15.1 log particles/mL (13.7 – 16.3) vs 10.5 (9.4 – 11.6); p<0.0001]. Amongst leucocyte derived CMPs, CD66b+ and CD16+ CD14- were 1.4-fold and 1.5-fold higher respectively [median (IQR) CD66b: 11.9 log particles/mL (11.3 – 12.7) versus 8.8 (7.9 – 9.3), p<0.0001. CD16+CD14-: 13.1 (11.3 – 133.9) vs 8.7 (7.4 – 9.1), p<0.0001]. For CD14 positive CMPs, only those expressing tissue factor were significantly higher [1.4-fold; median (IQR) 10.1 log particles/mL (8.3 – 11.3) vs 7.0 (6.2 – 8.1), p<0.0001].

CMPs of endothelial and platelet origin associated closely with cfPWV [spearman rho: E-selectin+ 0.57, p<0.0001 and CD42a 0.56, p<0.0001; Table 3). CD16 positive CD14 negative CMPs also correlated significantly with cfPWV (spearman rho 0.69, p<0.0001).

**Table 3.** Correlations between microparticle subsets and cfPWV.

		Total	
Circulating Microparticle subset		Spearman’s rho	P value
**Endothelial**	PECAM+CD42a-	0.63	<0.0001
	Eselectin+	0.61	<0.0001
	PECAM+Eselectin-	-0.34	0.004
	PECAM+Eselectin+	0.55	<0.0001
	PECAM-Eselectin+	0.53	<0.0001
	ICAM+PECAM+	0.33	0.02
	ICAM+PECAM-	0.58	<0.0001
	VCAM+PECAM+	0.26	0.07
	VCAM+PECAM-	0.46	<0.001
**Leucocyte**	CD66b+PECAM-	0.39	<0.01
	CD14-CD16+	0.74	<0.0001
	CD14+CD16+	0.08	0.78
	CD14+CD16-	-0.06	0.85
**Platelet**	CD42a+ PECAM-	0.61	<0.0001

## Discussion

Here we show that total CMPs correlate closely with arterial stiffness and are markedly increased amongst people who present with HIV related advanced immune suppression in Malawi. Microparticles originating from endothelium and platelets were especially high amongst people with HIV and correlated strongly with arterial stiffness; suggesting that these pathways might be prioritised for future studies seeking to reduce inflammation driven cardiovascular risk amongst PLWH during ART initiation. 

Each log increase in CMPs showed a 20% increase in cfPWV. A relationship between total CMPs and activated T cells was also demonstrated, in keeping with our previous data suggesting a role for activated and exhausted T cells in increased arterial stiffness amongst PLWH ^5^. However, the association between CMPs and arterial stiffness was not reduced when HIV was added as a variable to the model, suggesting that other factors may be at play in the generation of CMPs in this Malawian population.

CMPs are induced in response to cellular stress in the form of activation, apoptosis or physical sheer stress ^24^. A similar case control study of 15 PLWH established on ART in the USA, showed significantly elevated levels of all types of microparticles compared to 15 participants without HIV infection ^22^, where microparticles from PLWH directly impaired endothelial cell function. A larger study of PLWH from the Czech Republic also found an increase in microparticles compared to participants without HIV, but found no difference amongst those on ART compared to those who were at their first presentation ^25^. However, none of these studies assessed the relationship between CMPs and arterial stiffness. By presenting associations with a validated physiological biomarker, we provide evidence for the use of CMPs as a potential clinically relevant tool to support characterisation of inflammation amongst PLWH.

As well as acting as biomarkers of inflammation, microparticles have been implicated in mediating inflammation induced pathogenesis. They may interact with endothelial cell surface adhesion molecules and cause endothelial damage through the production of nitrous oxide and pro-inflammatory cytokines ^26^. CMPs have been shown to transfer important material such as miRNA and lipids to other cells ^27^. In particular, the transfer of the CCR5 receptor to endothelial cells from leucocyte derived microparticles potentially renders them permissible to direct HIV infection ^28^. Circulating microparticles produced in response to HIV could lead to endothelial damage, and therefore increased risk of cardiovascular disease, through multiple mechanisms including direct endothelial adhesion and activation, transfer of cytotoxic viral proteins, and propagation of low level viral replication. 

We discovered that microparticles of endothelial and platelet origin are particularly expanded amongst PLWH in this low income SSA setting. Defining endothelial microparticles as CD51+, da Silva *et al.* previously reported endothelial microparticles 20 times higher in PLWH compared to people without HIV ^18^. Endothelial microparticles promote expression of adhesion molecules on the endothelial cell surface, generating thrombosis, platelet activation and recruitment of inflammatory cells ^21^. Platelet driven thrombosis in combination with endothelial activation may take advantage of a compromised endothelial barrier and lead to decreased elasticity and smooth muscle reactivity within the arterial wall ^29^. Inflammatory cytokine release in response to ongoing cellular recruitment, as well as from direct effects of the HIV virus, can also activate and increase the proliferation and migration of vascular smooth muscle cells, and thus induce arterial stiffness ^30^. Wheway *et al.* have previously found endothelial microparticles to form conjugates with T cell subsets, with increased binding to those cells that were pre-stimulated ^31^. Binding was VCAM and ICAM dependent and endothelial microparticles were able to stimulate *in vitro* proliferation of T cells.

Although this study is limited by a small sample size and its cross sectional design, we demonstrate quantification and characterisation of circulating microparticles to be clinically relevant and also confirm that this applies in the low income sub-Saharan Africa setting. We also elucidate the platelet – endothelial axis as an interesting pathway worthy of further investigation in inflammation during early ART. This cohort of people living with HIV had experienced advanced immune suppression and so factors other than the direct effect of HIV itself (e.g. CMV, TB, cryptococcal disease) may be contributing. Further, findings are not generalisable to cohorts with more robust CD4 counts and therefore this needs to be established.

Overall, the characterisation of microparticles in this study lends weight to a model where active and significant immune activation amongst people living with HIV is strongly related to endothelial damage and may involve both CD8 T cell and platelet activation. Further research should investigate whether CMPs might represent translational targets to reduce inflammation driven cardiovascular risk amongst people living with HIV.

## References

[1] Farahani M Mulinder H Farahani A : Prevalence and distribution of non-AIDS causes of death among HIV-infected individuals receiving antiretroviral therapy: a systematic review and meta-analysis. *Int J STD AIDS.* 2017;28(7):636–50. 10.1177/0956462416632428 26868158

[2] Kuller LH Tracy R Belloso W : Inflammatory and coagulation biomarkers and mortality in patients with HIV infection. *PLoS Med.* 2008;5(10):e203. 10.1371/journal.pmed.0050203 18942885 PMC2570418

[3] Friis-Møller N Sabin CA Weber R : Combination antiretroviral therapy and the risk of myocardial infarction. *N Engl J Med.* 2003;349(21):1993–2003. 10.1056/NEJMoa030218 14627784

[4] Triant VA Meigs JB Grinspoon SK : Association of C-reactive protein and HIV infection with acute myocardial infarction. *J Acquir Immune Defic Syndr.* 2009;51(3):268–73. 10.1097/QAI.0b013e3181a9992c 19387353 PMC2763381

[5] Kelly C Mwandumba HC Heyderman RS : HIV-Related Arterial Stiffness in Malawian Adults Is Associated With the Proportion of PD-1-Expressing CD8 ^+^ T Cells and Reverses With Antiretroviral Therapy. *J Infect Dis.* 2019;219(12):1948–1958. 10.1093/infdis/jiz015 30629187 PMC6534190

[6] Craik A Patel P Patel P : Challenges with targeted viral load testing for medical inpatients at Queen Elizabeth Central Hospital in Blantyre, Malawi. *Malawi Med J.* 2016;28(4):179–81. 10.4314/mmj.v28i4.6 28321282 PMC5348611

[7] DART Trial Team; Mugyenyi P Walker AS : Routine versus clinically driven laboratory monitoring of HIV antiretroviral therapy in Africa (DART): a randomised non-inferiority trial. *Lancet.* 2010;375(9709):123–31. 10.1016/S0140-6736(09)62067-5 20004464 PMC2805723

[8] Heikinheimo T Chimbayo D Kumwenda JJ : Stroke outcomes in Malawi, a country with high prevalence of HIV: a prospective follow-up study. *PLoS One.* 2012;7(3):e33765. 10.1371/journal.pone.0033765 22479439 PMC3315584

[9] Benjamin LA Corbett EL Connor MD : HIV, antiretroviral treatment, hypertension, and stroke in Malawian adults: A case-control study. *Neurology.* 2016;86(4):324–33. 10.1212/WNL.0000000000002278 26683649 PMC4776088

[10] Laurent S Alivon M Beaussier H : Aortic stiffness as a tissue biomarker for predicting future cardiovascular events in asymptomatic hypertensive subjects. *Ann Med.* 2012;44 Suppl 1:S93–7. 10.3109/07853890.2011.653398 22713154

[11] Laurent S Boutouyrie P Asmar R : Aortic stiffness is an independent predictor of all-cause and cardiovascular mortality in hypertensive patients. *Hypertension.* 2001;37(5):1236–41. 10.1161/01.hyp.37.5.1236 11358934

[12] Laurent S Briet M Boutouyrie P : Arterial stiffness as surrogate end point: needed clinical trials. *Hypertension.* 2012;60(2):518–22. 10.1161/HYPERTENSIONAHA.112.194456 22733473

[13] Laurent S Cockcroft J Van Bortel L : Expert consensus document on arterial stiffness: methodological issues and clinical applications. *Eur Heart J.* 2006;27(21):2588–605. 10.1093/eurheartj/ehl254 17000623

[14] Sima AV Stancu CS Simionescu M : Vascular endothelium in atherosclerosis. *Cell Tissue Res.* 2009;335(1):191–203. 10.1007/s00441-008-0678-5 18797930

[15] Doran AC Meller N McNamara CA : Role of smooth muscle cells in the initiation and early progression of atherosclerosis. *Arterioscler Thromb Vasc Biol.* 2008;28(5):812–9. 10.1161/ATVBAHA.107.159327 18276911 PMC2734458

[16] Vion AC Ramkhelawon B Loyer X : Shear stress regulates endothelial microparticle release. *Circ Res.* 2013;112(10):1323–33. 10.1161/CIRCRESAHA.112.300818 23536307

[17] Zwaal RFA Comfurius P Bevers EM : Surface exposure of phosphatidylserine in pathological cells. *Cell Mol Life Sci.* 2005;62(9):971–88. 10.1007/s00018-005-4527-3 15761668 PMC11924510

[18] da Silva EF Fonseca FA França CN : Imbalance between endothelial progenitors cells and microparticles in HIV-infected patients naive for antiretroviral therapy. *AIDS.* (London, England).2011;25(13):1595–601. 10.1097/QAD.0b013e32834980f4 21673561

[19] Mayne E Funderburg NT Sieg SF : Increased platelet and microparticle activation in HIV infection: upregulation of P-selectin and tissue factor expression. *J Acquir Immune Defic Syndr.* 2012;59(4):340–6. 10.1097/QAI.0b013e3182439355 22156911 PMC3299881

[20] Baker JV Huppler Hullsiek K Bradford RL : Circulating levels of tissue factor microparticle procoagulant activity are reduced with antiretroviral therapy and are associated with persistent inflammation and coagulation activation among HIV-positive patients. *J Acquir Immune Defic Syndr.* 2013;63(3):367–71. 10.1097/QAI.0b013e3182910121 23507662 PMC3683107

[21] López M San Román J Estrada V : Endothelial dysfunction in HIV infection--the role of circulating endothelial cells, microparticles, endothelial progenitor cells and macrophages. *AIDS Rev.* 2012;14(4):223–30. 23258297

[22] Hijmans JG Stockelman KA Garcia V : Circulating Microparticles Are Elevated in Treated HIV -1 Infection and Are Deleterious to Endothelial Cell Function. *J Am Heart Assoc.* 2019;8(4):e011134. 10.1161/JAHA.118.011134 30779672 PMC6405669

[23] Van Bortel LM Laurent S Boutouyrie P : Expert consensus document on the measurement of aortic stiffness in daily practice using carotid-femoral pulse wave velocity. *J Hypertens.* 2012;30(3):445–8. 10.1097/HJH.0b013e32834fa8b0 22278144

[24] Martin S Tesse A Hugel B : Shed membrane particles from T lymphocytes impair endothelial function and regulate endothelial protein expression. *Circulation.* 2004;109(13):1653–9. 10.1161/01.CIR.0000124065.31211.6E 15023873

[25] Snopkova S Matyskova M Havlickova K : Increasing procoagulant activity of circulating microparticles in patients living with HIV. *Med Mal Infect.* 2020;50(7):555–561. 10.1016/j.medmal.2019.09.013 31611134

[26] Khaddaj Mallat R Mathew John C Kendrick DJ : The vascular endothelium: A regulator of arterial tone and interface for the immune system. *Crit Rev Clin Lab Sci.* 2017;54(7–8):458–70. 10.1080/10408363.2017.1394267 29084470

[27] Diehl P Fricke A Sander L : Microparticles: major transport vehicles for distinct microRNAs in circulation. *Cardiovasc Res.* 2012;93(4):633–44. 10.1093/cvr/cvs007 22258631 PMC3291092

[28] Mack M Kleinschmidt A Brühl H : Transfer of the chemokine receptor CCR5 between cells by membrane-derived microparticles: a mechanism for cellular human immunodeficiency virus 1 infection. *Nat Med.* 2000;6(7):769–75. 10.1038/77498 10888925

[29] Brown RA Shantsila E Varma C : Epidemiology and pathogenesis of diffuse obstructive coronary artery disease: the role of arterial stiffness, shear stress, monocyte subsets and circulating microparticles. *Ann Med.* 2016;48(6):444–55. 10.1080/07853890.2016.1190861 27282244

[30] Mazzuca P Caruso A Caccuri F : HIV-1 infection, microenvironment and endothelial cell dysfunction. *New Microbiol.* 2016;39(3):163–73. 27704142

[31] Wheway J Latham SL Combes V : Endothelial microparticles interact with and support the proliferation of T cells. *J Immunol.* 2014;193(7):3378–87. 10.4049/jimmunol.1303431 25187656 PMC4170003

