# Supplementary material for: Circulating microparticles are increased amongst people presenting with HIV and advanced immune suppression in Malawi and correlate closely with arterial stiffness: a nested case control study
Source: Wellcome Open Res. 2022 Oct 17;6:264. Originally published 2021 Oct 13. [Version 2] doi: 10.12688/wellcomeopenres.17044.2 (PMC9577278; doi:10.12688/wellcomeopenres.17044.2)
Supplement: Peer review history

# Peer review: Reviewer response for version 2

**DOI:** 10.21956/wellcomeopenres.20445.r52870

**Published:** 2022-10-19

**Authors:** Wilson L. Mandala

**Affiliations:** 1 Malawi University of Science and Technology, Thyolo, Malawi

Having read the responses from the authors and the revised version of the manuscript, I would like to submit that I do not have any further comments to be made and I would recommend that this manuscript be unconditionally accepted for indexing without any further reservations from me.

Is the work clearly and accurately presented and does it cite the current literature?

Yes

If applicable, is the statistical analysis and its interpretation appropriate?

Yes

Are all the source data underlying the results available to ensure full reproducibility?

Yes

Is the study design appropriate and is the work technically sound?

Yes

Are the conclusions drawn adequately supported by the results?

Partly

Are sufficient details of methods and analysis provided to allow replication by others?

Partly

Reviewer Expertise:

NA

I confirm that I have read this submission and believe that I have an appropriate level of expertise to confirm that it is of an acceptable scientific standard.

# Peer review: Reviewer response for version 2

**DOI:** 10.21956/wellcomeopenres.20445.r52868

**Published:** 2022-10-17

**Authors:** Joseph Heskin

**Affiliations:** 1 Department of HIV, GUM, Chelsea and Westminster NHS Foundation Trust, London, UK

I have reviewed all responses to my original comments and am satisfied by the authors explanations or amendments.

Is the work clearly and accurately presented and does it cite the current literature?

Yes

If applicable, is the statistical analysis and its interpretation appropriate?

Yes

Are all the source data underlying the results available to ensure full reproducibility?

Yes

Is the study design appropriate and is the work technically sound?

Yes

Are the conclusions drawn adequately supported by the results?

Partly

Are sufficient details of methods and analysis provided to allow replication by others?

Partly

Reviewer Expertise:

HIV

I confirm that I have read this submission and believe that I have an appropriate level of expertise to confirm that it is of an acceptable scientific standard.

# Peer review: Reviewer response for version 1

**DOI:** 10.21956/wellcomeopenres.18825.r51610

**Published:** 2022-08-30

**Authors:** Siddharth Kogilwaimath

**Affiliations:** 1 The Harvard T.H. Chan School of Public Health, Harvard University, Boston, MA, USA; 2 Division of Infectious Diseases, Department of Medicine, University of Saskatchewan, Saskatoon, SK, Canada

Great work on this by Dr. Kelly and colleagues. Very interesting topic that is relevant in HIV medicine. I have left suggestions based on the headings:

**Introduction: **

- Well written overall. 

**Methods:**

- Study Cohort: Unclear why the data was collected at 2 weeks into ART initiation. The lack of baseline data should be explained clearly. If IRIS is a plausible mechanism, could the choice of baseline ART used influence the total CMP expression? I am not sure if there was access to any INSTI based regimens during 2014-2015. I suspect that DTG was not widely used at that time but if an INSTI based regimen was used, is there a link with those on INSTI regimens and higher CMPs?
- Study Cohort: I am afraid it was not easy to follow the second paragraph. I would re-write this and simplify it.
- Laboratory procedures: Would suggest including a sentence on whether there is an established standard for CMP staining/gating and cell surface immunophenotyping and if so, how/why did this study standard differ?

**Discussion:**

- "However, the association between CMPs and arterial stiffness was not reduced when HIV was added, suggesting that other factors may be at play in the generation of CMPs in this Malawian population." especially around "when HIV was added"- I presume to the logistic regression model but this should be clarified. 
- Would include what proportion of these PLWH had an active OI or malignancy related to advanced HIV as these could also affect CMPs I would imagine. I would at least include a comment on this in case data is unavailable or not collected and why? If data is available, I would consider including that.
- Consider including conclusion of an association between a biomarker and arterial stiffness based on data collected at a single time-point somewhere in the discussion. Was there longer term follow-up of the CMPs? Did it come down along with the arterial stiffness after 3 months on ART to suggest that it follows the same trajectory as the arterial stiffness based on prior studies? Did it come down as previously hypothesized to have been due to IRIS? Also, the sample size is extremely small and subject to confounding by indication with OIs/advanced HIV related malignancy. 

Is the work clearly and accurately presented and does it cite the current literature?

Yes

If applicable, is the statistical analysis and its interpretation appropriate?

Yes

Are all the source data underlying the results available to ensure full reproducibility?

Yes

Is the study design appropriate and is the work technically sound?

Yes

Are the conclusions drawn adequately supported by the results?

Partly

Are sufficient details of methods and analysis provided to allow replication by others?

Yes

Reviewer Expertise:

HIV, Infectious Diseases.

I confirm that I have read this submission and believe that I have an appropriate level of expertise to confirm that it is of an acceptable scientific standard, however I have significant reservations, as outlined above.

# Response

**Published:** 2022-09-30

**Authors:** Christine Kelly

**Affiliations:** UCD Dublin, Ireland

Great work on this by Dr. Kelly and colleagues. Very interesting topic that is relevant in HIV medicine. I have left suggestions based on the headings:

 Introduction:

 •            Well written overall.

 Methods:

 •            Study Cohort: Unclear why the data was collected at 2 weeks into ART initiation. The lack of baseline data should be explained clearly. If IRIS is a plausible mechanism, could the choice of baseline ART used influence the total CMP expression? I am not sure if there was access to any INSTI based regimens during 2014-2015. I suspect that DTG was not widely used at that time but if an INSTI based regimen was used, is there a link with those on INSTI regimens and higher CMPs?

**We thank the reviewer very much for the helpful comments.** **Study procedures were carried out to reduce initial burden on this clinically vulnerable population – for whom the priority was safely initiating ART. The ART regime at this time is laid out in the referenced JID paper (reference 5), but first line regimes at that time did not include DTG.**

 •            Study Cohort: I am afraid it was not easy to follow the second paragraph. I would re-write this and simplify it.

**This has been rewritten for clarity.**

 •            Laboratory procedures: Would suggest including a sentence on whether there is an established standard for CMP staining/gating and cell surface immunophenotyping and if so, how/why did this study standard differ?

**CMP is a research tool with no established standard for measurement as yet. The protocols contained within this paper were specifically developed by the authors.**

 Discussion:

 •            "However, the association between CMPs and arterial stiffness was not reduced when HIV was added, suggesting that other factors may be at play in the generation of CMPs in this Malawian population." especially around "when HIV was added"- I presume to the logistic regression model but this should be clarified.

**This has been clarified accordingly and with thanks.**

 •            Would include what proportion of these PLWH had an active OI or malignancy related to advanced HIV as these could also affect CMPs I would imagine. I would at least include a comment on this in case data is unavailable or not collected and why? If data is available, I would consider including that.

**The numbers of patients with a concurrent infection at time of recruitment are shown in Table 1. The possibility that such infections may be contributing to inflammation over and above HIV is acknowledged within the limitations paragraph of the discussion section.**

 •            Consider including conclusion of an association between a biomarker and arterial stiffness based on data collected at a single time-point somewhere in the discussion. Was there longer term follow-up of the CMPs? Did it come down along with the arterial stiffness after 3 months on ART to suggest that it follows the same trajectory as the arterial stiffness based on prior studies? Did it come down as previously hypothesized to have been due to IRIS? Also, the sample size is extremely small and subject to confounding by indication with OIs/advanced HIV related malignancy.

**The small sample size and possible confounding by OIs have been highlighted as limitations in the penultimate paragraph of the discussion, which has now been flagged more clearly as a limitation section. We have added an additional limitation around the cross sectional nature of the work.**

# Peer review: Reviewer response for version 1

**DOI:** 10.21956/wellcomeopenres.18825.r51608

**Published:** 2022-08-03

**Authors:** Joseph Heskin

**Affiliations:** 1 Department of HIV, GUM, Chelsea and Westminster NHS Foundation Trust, London, UK

This manuscript is well written and laid out. I note the following points which I feel can be easily resolved:

**Major Comments**

1. The cohort described in this paper are termed the SHIELD cohort and the paper reports that details of recruitment are in reference 5 (Kelly *et al.*, 2019). This paper refers to the REALITY trial and does not mention the SHIELD cohort. Please can the authors clarify if this is the same cohort. 
2. Please can the authors explain why cfPWV measurements were not taken prior to the initiation of ART and only 2 weeks after initiation. This would appear to be important if you are using this as a marker of ART induced vascular dysfunction.

**Minor Comments**

Introduction:

1. Final paragraph "previous studies.." - please consider removing 'in HIV' as this is covered by discussion PLWH.

Methods:

1. Please consider switching the statements around so it reads that ethical approval was granted prior to participants providing informed written consent.
2. "SHIELD participants with HIV..." - it is unclear what is meant by ordered. Please consider rewriting the paragraph as it is not clear how and why convenience samples were chosen.
3. Were participants matched by gender?

Results:

1. What is the relevance of the participants attained educational level?

Discussion:

1. It is not clear from the recruitment whether participants living with HIV had active infections at the time of recruitment. Given the immunocompromised state, is there a concern for active OI's contributing to platelet and endothelial dysfunction?

Is the work clearly and accurately presented and does it cite the current literature?

Yes

If applicable, is the statistical analysis and its interpretation appropriate?

Yes

Are all the source data underlying the results available to ensure full reproducibility?

Yes

Is the study design appropriate and is the work technically sound?

Yes

Are the conclusions drawn adequately supported by the results?

Partly

Are sufficient details of methods and analysis provided to allow replication by others?

Partly

Reviewer Expertise:

HIV

I confirm that I have read this submission and believe that I have an appropriate level of expertise to confirm that it is of an acceptable scientific standard, however I have significant reservations, as outlined above.

# Response

**Published:** 2022-09-30

**Authors:** Christine Kelly

**Affiliations:** UCD Dublin, Ireland

This manuscript is well written and laid out. I note the following points which I feel can be easily resolved:

 Major Comments

 1.           The cohort described in this paper are termed the SHIELD cohort and the paper reports that details of recruitment are in reference 5 (Kelly et al., 2019). This paper refers to the REALITY trial and does not mention the SHIELD cohort. Please can the authors clarify if this is the same cohort.

**We thank the reviewer for highlighting this point of confusion. A proportion of SHIELD participants were co-recruited to the REALITY trial, the details of which are outlined in the referenced paper. We did not specifically refer to the REALITY trial here as it was not relevant to the current analysis. However, we have added a sentence to the methods to make it clear that the participants were co-recruited.**

 2.           Please can the authors explain why cfPWV measurements were not taken prior to the initiation of ART and only 2 weeks after initiation. This would appear to be important if you are using this as a marker of ART induced vascular dysfunction.

**Study procedures were carried out at 2 weeks post ART initiation because this was a clinically vulnerable and immunosuppressed group pf patients. We therefore did not want to burden them with study procedures at the first ART visit in order to ensure understanding of clinical concepts.**

**A sentence clarifying this has been added to the manuscript.**

 Minor Comments

 Introduction:

 1.           Final paragraph "previous studies.." - please consider removing 'in HIV' as this is covered by discussion PLWH.

**We have added ‘in the field of’ to show highlight that CMPs have been studied elsewhere if the reader would like to consider these further.**

 Methods:

 1.           Please consider switching the statements around so it reads that ethical approval was granted prior to participants providing informed written consent.

**This has been amended accordingly and with thanks.**

 2.           "SHIELD participants with HIV..." - it is unclear what is meant by ordered. Please consider rewriting the paragraph as it is not clear how and why convenience samples were chosen.

**This paragraph has been adjusted to make the selection process clearer for the reader. **

1. Were participants matched by gender?

**Gender was not found to be significantly associated with arterial stiffness in previous analyses of this cohort and therefore was not used as a variable for matching.**

 Results:

 1.           What is the relevance of the participants attained educational level?

**Education level was included as an indicator of socio-economic status which has been shown to be associated with inflammation in PLWH in low-income settings.**

 Discussion:

 1.           It is not clear from the recruitment whether participants living with HIV had active infections at the time of recruitment. Given the immunocompromised state, is there a concern for active OI's contributing to platelet and endothelial dysfunction?

**The numbers of patients with a concurrent infection at time of recruitment are shown in Table 1. The possibility that such infections may be contributing to inflammation over and above HIV is acknowledged within the limitations paragraph of the discussion section.**

# Peer review: Reviewer response for version 1

**DOI:** 10.21956/wellcomeopenres.18825.r46515

**Published:** 2021-10-26

**Authors:** Wilson L. Mandala

**Affiliations:** 1 Malawi University of Science and Technology, Thyolo, Malawi

This current paper by Kelly *et al. *is based on a study which was primarily aimed at characterising circulating microparticles in HIV-infected individuals. It was also aimed at assessing the association between CMPs with risk to developing cardiovascular "complications"? "diseases"?

 The manuscript is well laid out and presents the results in an orderly manner. However, I have the following observations to make:

 The manuscript needs to be re-written as there are quite a number of typos and some hanging statements (more details provided under Minor comments).

**Major comments:**

1. The authors point out under Methods/Study Cohort that "full details of recruitment and methods are available in Kelly *et al.*, JID 2019". However, the JID paper refers to a cohort of participants recruited into the REALITY study whereas this manuscript is based on the SHIELD study. The authors need to clearly explain if the two are one and the same. If not, how do the two studies differ?
2. Linked to point number one, the aim of the work in the JID paper was to characterize the contribution of immune activation to arterial stiffness in "HIV-infected Malawian adults **initiating** ART with advanced immunosuppression, compared with that in HIV-uninfected adults, and to determine how this changes over time on ART.", suggesting that participants were recruited **before** they had initiated ART. In this paper the aim of the study is "to characterise CMPs in HIV[-infected] individuals (who are recruited at what stage?). It is important to state at what stage of the HIV infection the study participants are recruited. This is only mentioned in the Abstract but **not **in the main text.
3. The aim also states: "to assess their [CMPs?] relationship with cardiovascular risk and elucidate pathways that might be involved in "heightened" inflammation during early ART". The results however only provide:
  3.1 association between absolute CMP counts, HIV-infection and arterial stiffness and
  3.2 association between CMP subsets, HIV and arterial stiffness.
4. There are no sets of results showing the direct relationship between CMPs with risk to develop cardiovascular complications. Therefore it would be better to rephrase the aim and link it with what the results are showing. Similarly, no potential pathways possibly involved in increased inflammation during early ART stages are provided and therefore this too should be rephrased or removed.
5. In the Discussion section the authors need to include the possible limitations of the study.

**Minor comments:**

 1. In the Abstract consider saying:

 Background: “We investigated whether circulating microparticle (CMP) subsets were elevated in individuals presenting with [untreated? ART-naïve? HIV] or HIV infection who had just commenced ART”. The group of study participants needs to be clearly defined right from the start.

 The Method section in the Abstract should read: “We characterized the microparticle and carotid femoral pulse wave velocity of ART-naïve adults with a new HIV diagnosis and CD4 [count] <100 cells/uL two weeks post ART initiation”. Although the study cohort is characterized clearly in the abstract, this detail is missing in the main text Methods section.

 The last sentence in the Conclusion which starts: “Endothelial damage and platelet …” is rather vague and should be re-written.

Introduction:

1. “but the underlying pathogenic mechanisms” - of what specifically?
2. “15.6 among those within the first 6 months of ART…” - please revisit the sentence and consider re-writing it
3. “The increase in arterial stiffness previously identified ... .”- please consider re-writing.
4.  “Previous studies in [humans infected with?] HIV… " - please consider changing as proposed.
5. Ethical considerations: “Ethical approval for the study was obtained from College of Medicine Research and Ethics Committee (COMREC) of the University of Malawi (P.09/13/1464) and from the University of Liverpool Research and Ethics Committee (UoL000996). Written informed consent was obtained from each participant before taking part in the study.
6. A [10-ml?] venous blood sample was collected in [EDTA tubes?] two weeks after ART initiation. [After centrifuging?] plasma was extracted and stored [at -86 ^o^C?] until the day of analysis. 
7. Please consider revising the entire paragraph which starts “SHIELD participants [infected with HIV?] were ordered [arranged? Ranked?] ... ” - the entire paragraph does not make sense.
8. Under Statistics, please consider changing “…0.003 was used as significant” - it doesn’t make sense.
9. Explain briefly how the agitation of the plates was done.
10. “350uL of each FACS tube was acquired” - please change to “350uL contents? Of each FACS tube was …”.
11. Under Cell surface Immunophenotyping consider starting the sentence with “T cell subsets surface Immunophenotyping …”.

Results:

1. What's the significance of the “education level of at least primary school completion"?
2. Please consider replacing “relationship” with “association”.
3. Please consider “Participants [infected?] with HIV”.
4. Table 1 - please consider presenting the Median results first on their own and have the Frequency results grouped together.
5. Figure 2-  please consider including the rho values and the p value within the figure.
6. Figure 3 - it might be worthwhile including lines showing which two groups are being compared. E.g line between the dots for No HIV vs HIV.

Discussion:

1. The whole Discussion section sounds a bit “casually written” and could do with a bit of re-writing.
2. Specify that the immune suppression in the third line is HIV-infection related.
3. The sentence which starts “However, the association between CMPs and arterial stiffness was not reduced ... ” should be considered for revision as its not clear.
4. There is need for a reference at the end of the sentence “As well as acting as biomarkers of inflammation…pathogenesis”.
5. Limitations of the study to be included in the Discussion section.

Is the work clearly and accurately presented and does it cite the current literature?

Yes

If applicable, is the statistical analysis and its interpretation appropriate?

Yes

Are all the source data underlying the results available to ensure full reproducibility?

Yes

Is the study design appropriate and is the work technically sound?

Yes

Are the conclusions drawn adequately supported by the results?

Partly

Are sufficient details of methods and analysis provided to allow replication by others?

Partly

Reviewer Expertise:

Immunology

I confirm that I have read this submission and believe that I have an appropriate level of expertise to confirm that it is of an acceptable scientific standard, however I have significant reservations, as outlined above.

## References

[1] : HIV-Related Arterial Stiffness in Malawian Adults Is Associated With the Proportion of PD-1–Expressing CD8+ T Cells and Reverses With Antiretroviral Therapy. *The Journal of Infectious Diseases* .2019;219(12) : 10.1093/infdis/jiz015 1948-1958 10.1093/infdis/jiz015 30629187PMC6534190

# Response

**Published:** 2022-09-30

**Authors:** Christine Kelly

**Affiliations:** UCD Dublin, Ireland

This current paper by Kelly et al. is based on a study which was primarily aimed at characterising circulating microparticles in HIV-infected individuals. It was also aimed at assessing the association between CMPs with risk to developing cardiovascular "complications"? "diseases"?

**We would think to thank the reviewer for their thoughtful and thorough comments. A sentence has been added to the introduction to provide clarity around the fact that arterial stiffness is a physiological marker of cardiovascular disease, closely correlated with future cardiovascular events (as evidenced in the referenced articles).**

 The manuscript is well laid out and presents the results in an orderly manner. However, I have the following observations to make:

 The manuscript needs to be re-written as there are quite a number of typos and some hanging statements (more details provided under Minor comments).

**We thank the reviewer for the suggestion and have amended throughout.**

 Major comments:

 1.           The authors point out under Methods/Study Cohort that "full details of recruitment and methods are available in Kelly et al., JID 2019". However, the JID paper refers to a cohort of participants recruited into the REALITY study whereas this manuscript is based on the SHIELD study. The authors need to clearly explain if the two are one and the same. If not, how do the two studies differ?

**The details of co-recruitment between SHIELD and REALITY are detailed in the referenced 5, and although the REALITY aspect of the recruitment is not relevant to the analyses presented in this current paper, we appreciate where confusion may arise and have added a sentence to the methods to help clarify this.**

 2.           Linked to point number one, the aim of the work in the JID paper was to characterize the contribution of immune activation to arterial stiffness in “HIV-infected Malawian adults initiating ART with advanced immunosuppression, compared with that in HIV-uninfected adults, and to determine how this changes over time on ART.”, suggesting that participants were recruited before they had initiated ART. In this paper the aim of the study is “to characterise CMPs in HIV[-infected] individuals (who are recruited at what stage?). It is important to state at what stage of the HIV infection the study participants are recruited. This is only mentioned in the Abstract but not in the main text.

**This point has been clarified in the main text to match the abstract.**

 3.           The aim also states: “to assess their [CMPs?] relationship with cardiovascular risk and elucidate pathways that might be involved in “heightened” inflammation during early ART”. The results however only provide:

 3.1 association between absolute CMP counts, HIV-infection and arterial stiffness and

 3.2 association between CMP subsets, HIV and arterial stiffness.

**We have added a clarification to the aims as stated in the final sentence of the introduction to show that arterial stiffness is measuring cardiovascular risk.**

**CMP subsets demonstrate different pathways that may be involved in inflammation by characterising the origins of microparticles, which are produced in response to stress and inflammation. This process is referred to in the first sentences of the fourth paragraph of the introduction.**

 4.           There are no sets of results showing the direct relationship between CMPs with risk to develop cardiovascular complications. Therefore it would be better to rephrase the aim and link it with what the results are showing. Similarly, no potential pathways possibly involved in increased inflammation during early ART stages are provided and therefore this too should be rephrased or removed.

**Throughout the manuscript we refer to cardiovascular risk, which is measured by arterial stiffness as a physiological marker for cardiovascular events. Much like we would use risk scores, such as the Framingham risk score to measure risk of cardiovascular events. Therefore, when we refer to cardiovascular risk, this is being measured by arterial stiffness. The relationship between arterials stiffness and cardiovascular events is explore in more detailed within the referenced articles. We have added clarifications to the introduction and methods to explain this more clearly.**

**Inflammatory pathways were assessed by characterised microparticle subsets. The most important subsets were found to be of endothelial and platelet origin, as highlighted in the discussion.**

 5.           In the Discussion section the authors need to include the possible limitations of the study.

**The limitations are described in the penultimate paragraph of the discussion. They include:**

- **Small convenience sample**
- **Cohort living with advanced HIV and risk of unmeasured confounders**
- **Limited generalisability**

**We have added clarification to the start of the paragraph to flag more clearly that it deals with study limitations.**

 Minor comments:

 1. In the Abstract consider saying:

 Background: “We investigated whether circulating microparticle (CMP) subsets were elevated in individuals presenting with [untreated? ART-naïve? HIV] or HIV infection who had just commenced ART”. The group of study participants needs to be clearly defined right from the start.

 The Method section in the Abstract should read: “We characterized the microparticle and carotid femoral pulse wave velocity of ART-naïve adults with a new HIV diagnosis and CD4 [count] <100 cells/uL two weeks post ART initiation”. Although the study cohort is characterized clearly in the abstract, this detail is missing in the main text Methods section.

**We have rephrased the abstract to make sure the population is clear from the start and that the methods section reads more clearly. We have maintained the passive voice for consistency throughout.**

 The last sentence in the Conclusion which starts: “Endothelial damage and platelet …” is rather vague and should be re-written.

**This sentence has been rewritten accordingly.**

 Introduction:

 1.           “but the underlying pathogenic mechanisms” - of what specifically?

**The underlying pathogenic mechanisms refers to the relationship between heightened inflammation and vascular dysfunction in people living with HIV.**

 2.           “15.6 among those within the first 6 months of ART…” - please revisit the sentence and consider re-writing it

**This sentence has been rewritten accordingly and with thanks.**

 3.           “The increase in arterial stiffness previously identified ... .”- please consider re-writing.

**This sentence has been rewritten accordingly and with thanks.**

 4.           “Previous studies in [humans infected with?] HIV… " - please consider changing as proposed.

**This sentence has been clarified; reference to these cohorts being people living with HIV (PLWH) is provided later in the sentence.**

 5.           Ethical considerations: “Ethical approval for the study was obtained from College of Medicine Research and Ethics Committee (COMREC) of the University of Malawi (P.09/13/1464) and from the University of Liverpool Research and Ethics Committee (UoL000996). Written informed consent was obtained from each participant before taking part in the study.

**This section has been added.**

 6.           A [10-ml?] venous blood sample was collected in [EDTA tubes?] two weeks after ART initiation. [After centrifuging?] plasma was extracted and stored [at -86oC?] until the day of analysis.

**These details have been outlined within the microparticle isolation section.**

 7.           Please consider revising the entire paragraph which starts “SHIELD participants [infected with HIV?] were ordered [arranged? Ranked?] ... ” - the entire paragraph does not make sense.

**Clarifications have been made to this paragraph which we hope will make it clearer for the reader.**

 8.           Under Statistics, please consider changing “…0.003 was used as significant” - it doesn’t make sense.

**This sentence has been added to for clarity.**

 9.           Explain briefly how the agitation of the plates was done.

**This has been added to the methods.**

 10.         “350uL of each FACS tube was acquired” - please change to “350uL contents? Of each FACS tube was …”.

**This has been changed accordingly.**

 11.         Under Cell surface Immunophenotyping consider starting the sentence with “T cell subsets surface Immunophenotyping …”.

**Both T cell subsets and monocyte subsets were assessed as detailed in the remainder of the sentence.**

 Results:

1. What's the significance of the “education level of at least primary school completion"?

**Education level gives an indication of the socio-economic status of the participants. Socio-economic status may be associated with risk of inflammation.**

 2.           Please consider replacing “relationship” with “association”.

**We like the word relationship to describe this analysis.**

 3.           Please consider “Participants [infected?] with HIV”.

**This has been changed to PLWH as standard terminology.**

 4.           Table 1 - please consider presenting the Median results first on their own and have the Frequency results grouped together.

**We feel that making two separate tables for characteristics may affect the fluidity of the results section for the reader.**

 5.           Figure 2-  please consider including the rho values and the p value within the figure.

**We have added the rho and p values to the figure legend.**

 6.           Figure 3 - it might be worthwhile including lines showing which two groups are being compared. E.g line between the dots for No HIV vs HIV.

**Due to the large number of graphs in this figure it is difficult to add lines without making the figure over crowded.**

 Discussion:

 1.           The whole Discussion section sounds a bit “casually written” and could do with a bit of re-writing.

**We are happy with the tone of the discussion, aimed for the general reader. We have addressed the reviewer’s specific queries regarding the discussion section below.**

 2.           Specify that the immune suppression in the third line is HIV-infection related.

**The word related has been inserted for clarity.**

 3.           The sentence which starts “However, the association between CMPs and arterial stiffness was not reduced ... ” should be considered for revision as its not clear.

**This sentence has been clarified.**

 4.           There is need for a reference at the end of the sentence “As well as acting as biomarkers of inflammation…pathogenesis”.

**The references for the different mechanisms in which microparticles have been implicated in inflammation appear in the subsequent sentences that detail the individual mechanisms.**

 5.           Limitations of the study to be included in the Discussion section.

**The penultimate paragraph deals with limitations as outlined above, and has now been flagged more clearly as a limitations section.**
